# Biological Activity of Blackcurrant Extracts (*Ribes nigrum* L.) in Relation to Erythrocyte Membranes

**DOI:** 10.1155/2014/783059

**Published:** 2014-01-16

**Authors:** Dorota Bonarska-Kujawa, Sylwia Cyboran, Romuald Żyłka, Jan Oszmiański, Halina Kleszczyńska

**Affiliations:** ^1^Department of Physics and Biophysics, Wrocław University of Environmental and Life Sciences, Norwida 25, 50-375 Wrocław, Poland; ^2^Department of Fruits, Vegetable and Cereals Technology, Wrocław University of Environmental and Life Sciences, Chełmońskiego 37/41, 51-630 Wrocław, Poland

## Abstract

Compounds contained in fruits and leaves of blackcurrant (*Ribes nigrum* L.) are known as agents acting preventively and therapeutically on the organism. The HPLC analysis showed they are rich in polyphenol anthocyanins in fruits and flavonoids in leaves, that have antioxidant activity and are beneficial for health. The aim of the research was to determine the effect of blackcurrant fruit and leaf extracts on the physical properties of the erythrocyte membranes and assess their antioxidant properties. The effect of the extracts on osmotic resistance, shape of erythrocytes and hemolytic and antioxidant activity of the extracts were examined with spectrophotometric methods. The FTIR investigation showed that extracts modify the erythrocyte membrane and protect it against free radicals induced by UV radiation. The results show that the extracts do not induce hemolysis and even protect erythrocytes against the harmful action of UVC radiation, while slightly strengthening the membrane and inducing echinocytes. The compounds contained in the extracts do not penetrate into the hydrophobic region, but bind to the membrane surface inducing small changes in the packing arrangement of the polar head groups of membrane lipids. The extracts have a high antioxidant activity. Their presence on the surface of the erythrocyte membrane entails protection against free radicals.

## 1. Introduction

Blackcurrant (*Ribes nigrum* L.) is a shrub commonly grown in various parts of the world of temperate climate. Its tasteful fruits are a rich source of vitamin C and other health beneficial substances such as: routine, organic acids, pectins, micro- and macronutrients and essential oils [1].

Blackcurrant fruits contain polyphenolic substances with antioxidant, antimicrobial, antiviral, and antibacterial properties [2–6]. Owing to these properties, polyphenols protect and support many functions of organs and systems and in particular the digestive [3, 7], nervous [8], and circulatory [9, 10] systems. In cells cultured in vitro, polyphenols exhibit anticancer activity, inhibiting the multiplication and growth of cancer cells by inducing apoptosis in them [11, 12]. Anthocyanins, in particular derivatives of cyanidin and delphinidin, which are the main polyphenols in fruit extract, are used in the treatment of eye defects and diseases of the eye [13, 14].

Contained in the leaves of blackcurrant, quercetin derivatives, as indicated in many studies, have a range of activities, including antimicrobial, anti-inflammatory, antiviral, antitoxic, antiseptic, and antioxidant effects, and are supposed to support the treatment of cancers [15–19]. Very good antioxidant properties of quercetin-3-O-glucoside in relation to biological membranes were showed in previous studies by the authors [20, 21].

For oxidation and destruction of biological systems are responsible high concentrations of free radicals that are formed either during metabolic processes or as a result of exposure to UV radiation. A conspicuous and important place of attack of free radicals is the cell membrane. Oxidation of its components and, in particular, the membrane lipids by free radicals causes disorder in the structure and function of the cell membrane, which leads to pathological changes in the organism. Development of many dangerous diseases linked directly with peroxidation of membrane lipids can be prevented by providing the organism with natural antioxidants, which are polyphenolic substances contained in different parts of the plant. Their protective effects, as scavengers of free radicals, depend on both the number of hydroxyl groups in the polyphenolic molecule as well as the number of molecules associated with the membrane. They protect the red blood cell membrane against oxidation and hemolysis induced by free radicals [22–26]. As indicated in our previous studies, the effectiveness of certain flavonoids, anthocyanins in particular, is much greater than the activity of vitamin E and its synthetic counterpart Trolox [20, 27].

High antioxidant activity of plant extracts and their ingredients was shown in numerous studies conducted around the world [4, 16, 17, 24]. The authors of many works consider the effective protection of biological membranes against oxidation dependent on the capabilities of the polyphenolic substances binding to membranes [22–26]. Due to the amphiphilic nature of polyphenols, it can be expected that they incorporate, mainly due to their structural similarity, at different depths into the lipid phase of biological membranes, changing their properties to varying degrees. However, there is little work on the impact of plant extracts and polyphenolic compounds on the properties of biological systems, and in particular of biological membranes. An important issue, therefore, seems to be the relationship between the high antioxidant activity of plant polyphenols in relation to biological membranes and the extent of physical changes that these substances induce in membranes. Such studies have been carried out to a minor extent for selected plant extracts in relation to membrane of erythrocytes and lipid membrane models [23, 24, 26, 28–31].

The present study aimed to determine the antioxidative activity of extracts from the fruit (BCF) and leaves (BCL) of blackcurrant, with respect to the biological membrane, and in particular in relation to membrane lipids. Given the rich polyphenolic composition of blackcurrant extracts, one could expect an effective protection of the membranes against oxidation-inducing agents. The research was conducted with respect to the membrane of erythrocytes, which was treated as an example and model of the biological membrane. Antioxidative activity of the extracts was determined on the basis of fluorimetric studies, with oxidation of erythrocyte membranes induced by UVC radiation and the AAPH compound. Also, the influence of extracts from fruits and leaves of blackcurrant on the properties of the erythrocyte membrane was examined, taking into account their possible negative impact. In particular, the hemolytic activity of the extracts and their impact on osmotic resistance of erythrocytes were assayed spectrophotometrically. Erythrocyte shapes were also examined in the presence of extracts, using the optical and electron microscopes. Using the Fourier transform infrared spectroscopy (FTIR) method, it was specified the location in the membrane of red blood cells of the phenolic substances contained in the extracts and their impact on its degree of hydration. Also, the protective effect of the extracts towards changes in the membrane of erythrocytes due to its exposure to UVC radiation was determined.

## 2. Materials and Methods

### 2.1. Materials

Plant extracts of fruits and leaves of blackcurrant (*Ribes nigrum *L.) were obtained from the Department of Fruit, Vegetable, and Cereal Technology, Wroclaw University of Environmental and Life Sciences. The percent content of polyphenols in the extracts was determined with liquid chromatography (HPLC).

The studies were conducted on pig erythrocytes and isolated erythrocyte membranes (ghosts), which were obtained from fresh blood using the Dodge method [32]. The content of erythrocyte membranes in the samples was determined on the basis of protein concentration, which was assayed using the Bradford method [33]. The choice of pig erythrocytes was dictated by the fact that this cell's percentage share of lipids is closest to that of the human erythrocyte, and the blood was easily available.

The fluorescent probe 3-(p-(6-phenyl)-1.3.5-hexatriene) propionic acid (DPH-PA) was purchased from Molecular Probes, Eugene, Oregon, USA. The oxidation inductor 2,2′-diazobis (2-amidinopropane) dihydrochloride (AAPH) and Trolox was purchased from Sigma-Aldrich, Steinheim, Germany.

Erythrocyte membranes were irradiated with UVC radiation from bactericidal lamp (3,5 mW/cm^2^).

### 2.2. Methods

#### 2.2.1. HPLC-DAD and UPLC-ESI/MS

Polyphenols were isolated from leaves and fruits by extraction with water containing 200 ppm of SO_2_, the ratio of solvent to leaves or fruits being 3 : 1. The extract was absorbed on Purolite AP 400 (UK) for further purification. The polyphenols were then eluted out with 80% ethanol, concentrated, and freeze-dried. The percent content of polyphenols in individual preparations was determined by means of liquid chromatography HPLC and UPLC method. Phenolic compounds were identified with the HPLC/DAD method and the method of UPLC/ESI/MS analysis described in papers [34].

#### 2.2.2. Antioxidant Activity of Extract

The DPH-PA probe was used in the fluorimetric experiments. Erythrocyte membranes with and without (control) addition of extracts were suspended in a phosphate buffer of pH 7.4 and UVC irradiated or treated with the chemical oxidation compound AAPH for 30 min. Free radicals, released in the process of membrane lipids irradiation, cause quenching of DPH-PA fluorescence, decreasing the fluorescence intensity. As a measure of the extent of lipid oxidation was assumed relative fluorescence, that is, the ratio of an UVC-irradiated or AAPH oxidized probe fluorescence to the initial fluorescence of the probe. Here, as a control was assumed the relative fluorescence of an erythrocyte membranes suspension that contained the DPH-PA probe, oxidized with UVC or AAPH compound, while the blank was the relative fluorescence of a suspension of the same concentration but not oxidized.

A spectrofluorimeter (Carry Eclipsce, Varian) was used to measure free radicals concentration in the samples. Excitation and emission wavelengths were *λ*_ex_ = 364 nm and *λ*_em_ = 430 nm. The measure of lipid oxidation was the relative change of fluorescence intensity *F*/*F*_0_, where *F*_0_ is the initial fluorescence and *F* the one measured during an oxidation procedure described in a paper [35]. The percentage of lipid oxidation inhibition was calculated from the following formula:

(1) $$\begin{array}{c} \text{inhibition}\%=\frac{\left(F_{x}-F_{u}\right)}{\left(F_{k}-F_{u}\right)}\cdot 100\%, \end{array}$$

where *F*_*x*_ is relative fluorescence of an UVC irradiated sample, or oxidized by AAPH, for 30 min in the presence of the compounds, *F*_*u*_ is relative fluorescence of the control sample, oxidized by AAPH or UVC irradiated, measured after 30 min, and *F*_*k*_ is relative fluorescence of the blank sample, not subjected to oxidation procedure, measured after 30 min.

The results of the assay were expressed in reference to Trolox, standard antioxidant.

#### 2.2.3. Hemolytic Activity of Extracts and Osmotic Resistance of Erythrocytes

The hemolytic experiments were conducted on fresh, heparinized blood. For washing the erythrocytes, and in the experiments, an isotonic phosphate solution of pH 7.4 (131 mM NaCl, 1.79 mM KCl, 0.86 mM MgCl_2_, 11.79 mM Na_2_HPO_4_  × 2H_2_O, and 1.80 mM Na_2_H_2_PO_4_  × H_2_O) was used. Full blood was centrifuged for 3 min, 1251 g at 4°C to remove the plasma and leucocytes. Upon removing from plasma, the erythrocytes were washed four times in phosphate solution and then incubated in the same solution but containing proper amounts of the compounds studied. The modification was conducted at 37°C for 1 h, each sample containing 10 mL of erythrocyte suspension of 2% hematocrit was stirred continuously. After modification 1 mL samples were taken, centrifuged and the supernatant assayed for hemoglobin content using a UV-Vis spectrophotometer (Cary 300 Bio, Varian) at 540 nm wavelength. Hemoglobin concentration in the supernatant, expressed as percentage of hemoglobin concentration of totally hemolyzed cells, was assumed as the measure of the extent of hemolysis.

For osmotic resistance, upon removing from plasma the erythrocytes were washed three times with a cool (ca. 4°C), 310 mosm PBS isotonic solution. Next, a 2% red cells suspension containing plant extracts of 0.01 mg/mL concentration was prepared and left for 1 h at 37°C with continuous stirring. After this modification the suspension of erythrocytes was centrifuged for 15 min at room temperature in order to remove the cells from the extract solution. From the cell sediment were taken 100 *μ*L samples of the extract-modified cells and suspended in test tubes containing NaCl solutions of 0.5–0.86% concentration and to an isotonic (0.9%) NaCl solution. In solutions of the same concentrations were also suspended unmodified red blood cells that constituted the control for osmotic resistance determinations. Then, the suspension was stirred and centrifuged under the above stated conditions. After that the percentage of hemolysis was measured with a spectrophotometer at *λ* = 540 nm. On the basis of the results obtained, the relation was determined between the percentage of hemolysis and NaCl concentration in the solution. Next, using the obtained plots, the NaCl percent concentrations (C_50_) that caused 50% hemolysis were found. The C_50_ values were taken as a measure of osmotic resistance. If a determined sodium chloride concentration is higher than that of control cells, the osmotic resistance of the erythrocytes is regarded to be lower, and vice versa.

#### 2.2.4. Erythrocyte Shapes

For investigation with the optical microscope, the red cells separated from plasma were washed four times in saline solution and suspended in the same solution but containing 0.01 and 0.1 mg/mL of BCL and BCF extracts, respectively. Hematocrit of the erythrocytes in the modification solution was 2%, the modification lasting 1 h at 37°C. After modification the erythrocytes were fixed with a 0.2% solution of glutaraldehyde. After that the red cells were observed under a biological optical microscope (Nikon Eclipse E200) equipped with a digital camera. The photographs obtained made it possible to count erythrocytes of various shapes and then percent share of the two basic forms (echinocytes and stomatocytes) in a population of ca. 800 cells was determined. The individual forms of erythrocyte cells were assigned morphological indices according to the Bessis scale [36], which for stomatocytes assume negative values from −1 to −4 and for echinocytes-positive from 1 to 4.

For investigation with the electron microscope, the red cells separated from plasma were washed four times in saline solution and suspended in the same solution but containing 0.1 mg/mL of the blackcurrant leaf (BCL) and fruit (BCF) extracts. Hematocrit of the erythrocytes in the modification solution was 2%, the modification lasting 1 h at 37°C. After modification the erythrocytes were fixed for 48 h in 2.5% solution of glutaraldehyde. After that the preparations were washed in phosphate buffer for 20 min, and then the material was dehydrated in a rising series of acetone concentrations (30, 50, 60, 70, 80, 90 and 100%). Each sample was washed for 15 min in an appropriate concentration and the material remain in pure acetone for 30 min. Next, the erythrocytes were dried for 12 h at room temperature. Erythrocytes thus prepared were deposited on object stages and subjected to X-ray microanalysis by means of an X-ray analyzer, Brucker AXS Quantax, collaborating with the ESPRIT ver. 1.8.2. program. Next, the samples were coated with gold using the Scancoat 6 (Edwards, London) sprinkler. The material ultrastructure was analyzed using a scanning microscope (EVO LS15 ZEISS) with SE1 detector, under high vacuum and accelerating voltage EHT = 20 kV.

#### 2.2.5. FTIR Investigation of Erythrocyte Membrane

Erythrocyte ghosts were washed three times in 0.9% NaCl solution. Next, the ghost suspension was incubated as 1 mL samples (150 *μ*L ghosts + 850 *μ*L physiological salt) for 24 h at 37°C. In that way the control sample with ghosts suspension was prepared, as well as samples with blackcurrant fruit and leaf extracts, obtaining 0.05 mg/mL concentration. After incubation, the samples were centrifuged for 15 min at 16000 g and IR spectra were recorded from a condensed membrane suspension. Tests were also conducted with samples containing erythrocyte membranes irradiated with UV, prepared in a similar way. After 24 h incubation with extracts, the ghost suspension was exposed to UV lamp for 1 h and then centrifuged and spectra taken in the IR range. The measurements were performed with a Thermo Nicolet 6700 MCT spectrometer, with ZnSe crystal, applied at room temperature. Each single spectrum was obtained from 128 records at 2 cm^−1^ resolution in the range 700–4000 cm^−1^. Preliminary elaboration of a spectrum was done using the EZ OMNIC v 8.0 program, also of the Thermo Nicolet firm. After filtering the noise out from the spectrum of the object studied, the spectrum of the NaCl solution was subtracted in order to remove the OH band of water and the base line was corrected.

## 3. Results

### 3.1. HPLC-DAD and UPLC-ESI/MS

Detailed quantitative and qualitative contents of phenolic compounds in the extracts from leaves and fruits of blackcurrant are given in Table 1.

The phenolic contents of blackcurrant leaves indicate that the dominant components in the leaves are quercetin derivatives, which constitute 80% of polyphenolic compounds in dry mass of extract. Very interesting though is the polyphenol composition of blackcurrant fruits, constituting over a half (56.14%) of total dry mass of extract.

**Figure 1:**
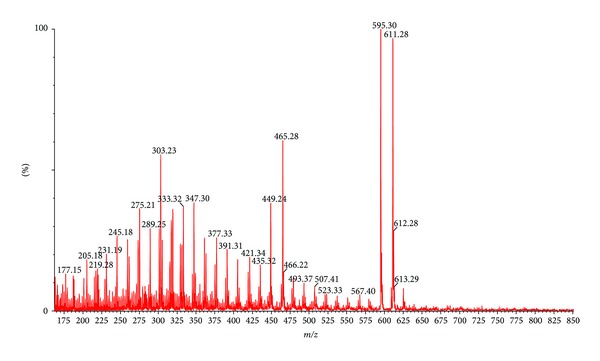
Direct injection ESI-MS of polyphenols in the blackcurrant fruit extract. Positively charged molecular ion are 449.24 cyanidin-3-O-glucoside, 465.28 delphinidin-3-O-glucoside, quercetin-3-O-glucoside and quercetin-3-O-galactoside, 595.30 cyanidin-3-O-rutinoside, 611.28 delphinidin-3-O-rutinoside and quercetin-3-O-rutinoside, and 625 petunidin-3-O-rutinoside.

**Figure 2:**
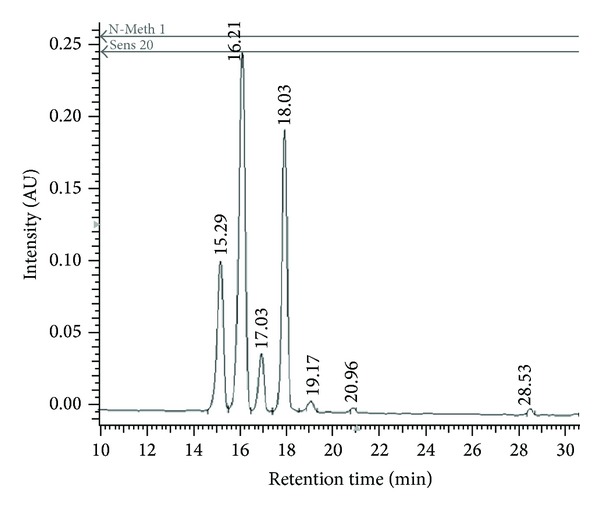
HPLC profile (520 nm) of blackcurrant extracts tr. 15.29 min delphinidin-3-O-glucoside, 16.21 min delphinidin-3-O-rutinoside, 17.03 min cyanidin-3-O-glucoside, 18.03 min cyanidin-3-O-rutinoside, and 19.17 min petunidin-3-O-rutinoside.

The blackcurrant fruit extract was analyzed by UPLC-ESI-MS and HPLC-DAD systems and is summarized in Table 1 and Figures 1 and 2. A total of 13 kinds of polyphenolic compounds found in blackcurrant fruit extract were identified and quantified. Two hydroxycinnamates were detected: chlorogenic and p-coumaroylquinic acids. The compounds that had a [M − H]^−^ at *m/z* 353 and 341 with *λ*_max_ 320 nm and 311 nm after a fragmentation yielded a caffeic and p-coumaric acid, respectively.

Anthocyanins, delphinidin-3-O-glucoside (peak tr. 15.29 min), delphinidin-3-O-rutinoside (peak tr. 16.21 min), cyanidin-3-O-glucoside (peak tr. 17.03 min), cyanidin-3-O-rutinoside (peak tr. 18.03 min), petunidin-3-O-rutinoside (peak tr. 19.17 min) (Figure 2), were identified on the basis of reference compounds, UV-Vis spectra, mass spectra, and the literature. Delphinidin and cyanidin were the two major anthocyanidins, commonly known in blackcurrant fruit [37]. The minor peak was identified as petunidin aglycone.

Furthermore, a total of 6 flavonol glycosides were detected (Table 1). Three quercetin derivatives were detected: quercetin-3-O-rutinoside, quercetin-3-O-galactoside and quercetin-3-O-glucoside. Peaks with (*R*_*t*_) 23.17 min, 23.77 min and 24.46 min had *λ*_max_ of 355 nm, respectively. All compounds had a fragmentation yielded a quercetin ion at *m/z* 303. Galactosides and glucosides have the same molar masses and mass spectra, and they differ according to their retention times only. Analogously, myricetin galactoside (*R*_*t*_) 20.71 min was assumed to eluate before myricetin glucoside (*R*_*t*_) 21.29 min. Myricetin-3-O-rutinoside (*R*_*t*_) 19.04 min was also identified in our samples, as defined in earlier investigations [38–40]. The anthocyanidin derivatives were the most predominant phenolic group found in blackcurrant fruit extract and constituted 54.49% weight of powder. Among identified anthocyanidin derivatives the most important compounds were delphinidin and cyanidin rutinoside (43.67%) The other compounds such as hydroxycinnamic acids and flavonol derivatives accounted total for 1.66% of blackcurrant fruit extract.

### 3.2. Antioxidant Activity of Extract

The investigation of antioxidative activity of BCF and BCL extracts were conducted on erythrocyte membranes, based on the extent of membrane lipids oxidation. The oxidation was induced with UVC and the AAPH compound. The extent of lipid oxidation was assayed fluorimetrically, based on the kinetics of DPH-PA probe quenching caused by free radicals that arose during oxidation.

The relative fluorescence for the studied concentrations of BCF and BCL extracts decreases with time of oxidation with UVC radiation, which shows that the degree of the lipid oxidation inhibition increases. Quenching of DPH-PA fluorescence in the presence of BCF and BCL extracts at different concentrations was also observed when lipid oxidation was induced in erythrocyte membranes with the chemical compound AAPH at 60 *μ*M.

**Figure 3:**
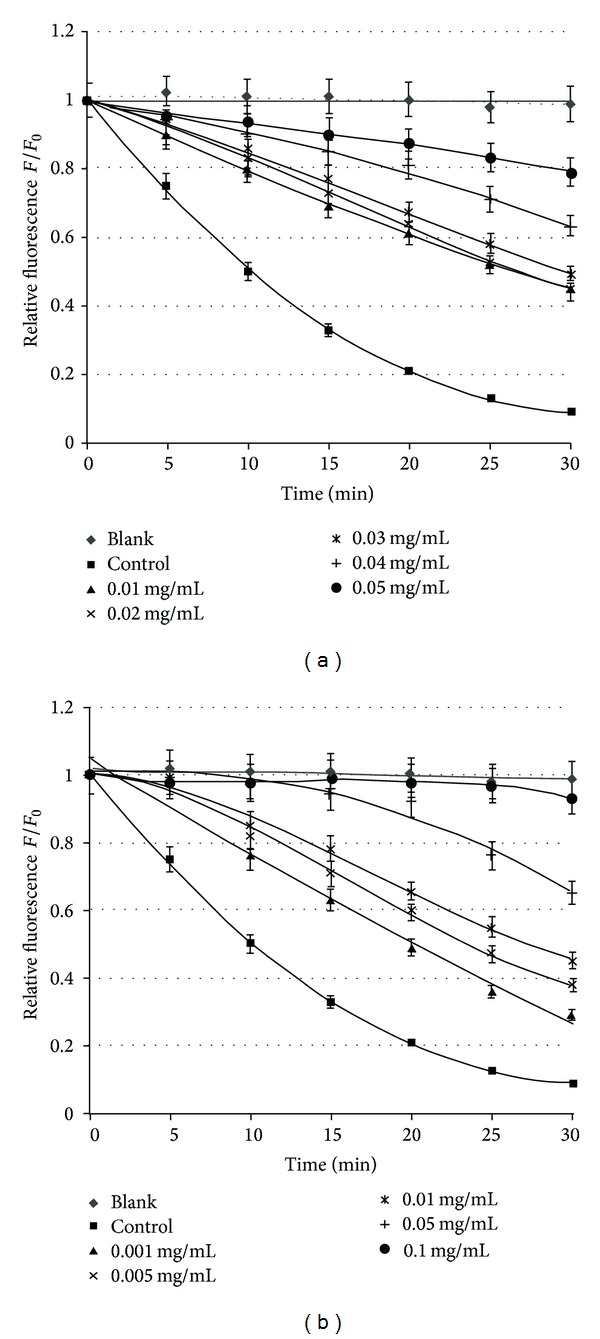
Relation between relative fluorescence intensity and time of UVC induced oxidation of erythrocyte membranes for control and test sample at different concentrations of blackcurrant extracts of leaves (a) and fruits (b).

Based on the kinetics of the oxidation curves obtained for various concentrations of both the extracts and compared with Trolox, the concentration responsible for 50% inhibition of membrane lipids oxidation (IC_50_) was found. The results from the fluorimetric method are given in Table 2 and Figure 3.

The results indicate that the extracts protect erythrocyte membrane lipids against free radicals induced with UVC and the AAPH compound. Extract concentration responsible for 50% protection of the lipids is comparable with that of Trolox in the case of AAPH and smaller when oxidation was UVC induced.

As indicated by the IC_50_ values, BCL and BCF extracts protect membrane lipids against oxidation. The results obtained have shown that BCL extract protect the erythrocyte membrane against UVC-induced oxidation better than BCF extract but worse than Trolox. For protecting membranes against AAPH-induced oxidation, the BCL and BCF extracts were equal in antioxidant activity to Trolox. Such results suggest that the reaction with free radicals depends on the oxidation inducer. In the case of photo-oxidation the percentage of inhibition and the kinetics of the process are slower, whereas in the case of the AAPH compound the process is much faster and efficient in inhibiting oxidation. The results indicate that polyphenols, mainly of the flavonoid group, contained in blackcurrant extract have good antioxidative properties, being more effective towards free radicals that arise in the process of AAPH-induced oxidation of erythrocyte membranes than UVC-induced one.

### 3.3. Hemolysis and Osmotic Resistance of Erythrocytes

The results on the effect of blackcurrant extracts on hemolysis of erythrocytes compiled in Table 3 testify that in the presence of BCF and BCL extracts red blood cells do not undergo increased hemolysis compared with unmodified cells, hemolysis with extract at 0.1 mg/mL not exceeding 5% in respect to the control probe. The control probe contained suspension of unmodified erythrocyte cells in phosphate buffer (pH 7.4).

**Figure 4:**
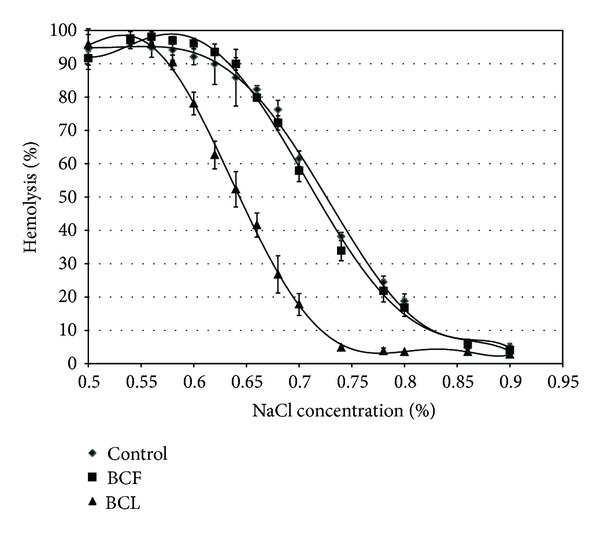
Percent of hemolysis of cells modified with blackcurrant leaf (BCL) and fruit (BCF) extract of 0.01 mg/mL concentration versus sodium chloride concentration.

In the studies of the effect of the extracts on erythrocyte osmotic resistance no significant differences between the degree of hemolysis in the control cells and those modified with BCF extract was found, in aqueous solutions of various concentrations of sodium chloride. Concentrations (C_50_) of sodium chloride, expressed as a percentage, determined for control blood cells and those modified with BCL and BCF extracts of 0.01 mg/mL concentration are C_50_ control—0.725%, BCL—0.644%, BCF—0.714%. These results indicate an increase in osmotic resistance of blackcurrant leaf extract modified cells, suggesting that the red blood cell membrane treated with BCL extract is less sensitive to changes in osmotic pressure (Figure 4).

### 3.4. Erythrocyte Shapes

**Figure 5:**
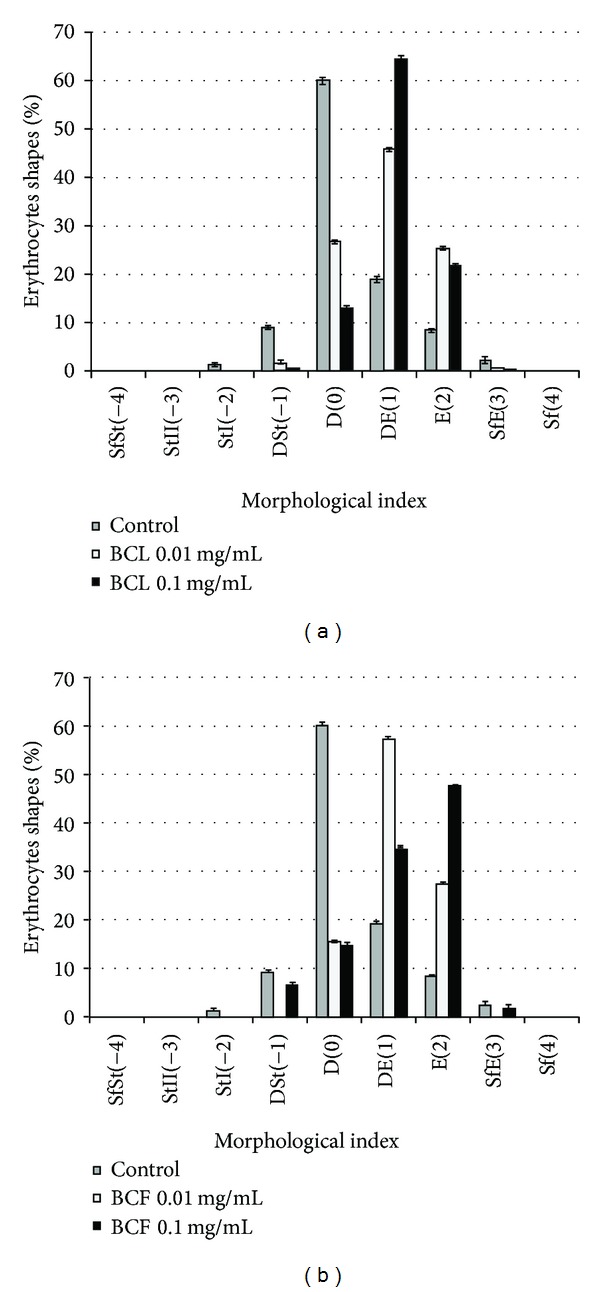
Percentage share of different shapes of erythrocytes induced by BCL and BCF extracts at 0.1 (black bar) and 0.01 (white bar) mg/mL concentration, control (grey bar). On the abscissa, there are morphological indices for the respective shapes of cells: spherostomatocytes (−4), stomatocytes II (−3), stomatocytes I (−2), discostomatocytes (−1), discocytes (0), discoechinocytes (1), echinocytes (2), spheroechinocytes (3), and spherocytes (4).

**Figure 6:**
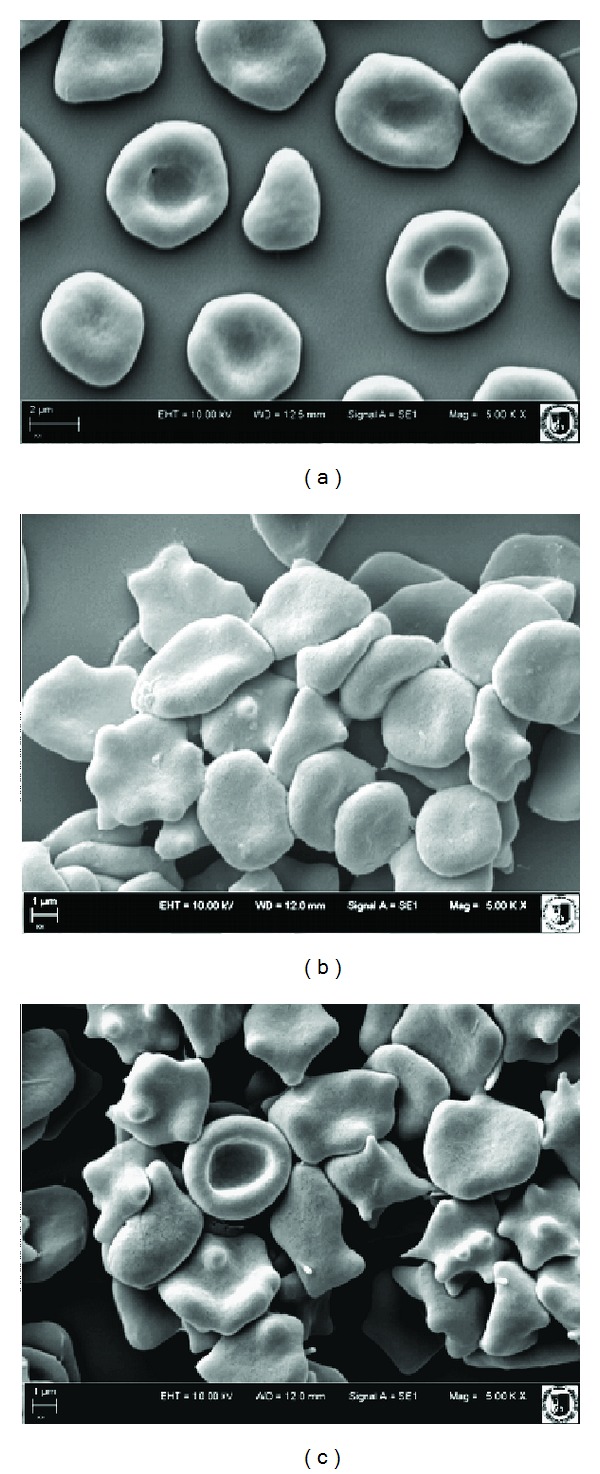
Shapes of unmodified erythrocytes (a) and modified with BCF (b) and BCL (c) at 0.1 mg/mL concentration, observed with electron microscope.

Figure 5 shows the percent share of the various forms of cells in a population of erythrocytes modified with BCL and BCF extracts of 0.1 and 0.01 mg/mL concentration. As seen in Figures 5(a), 5(b), and 6, these extracts induce mostly various forms of echinocytes, mainly discoechinocytes. It can thus be assumed that the blackcurrant extracts concentrate mainly in the outer monolayer of the erythrocyte membrane when inducing echinocytes, and practically do not permeate into the inner monolayer of the membrane [41–44].

The work [43, 44] showed that the formation of echinocytes occurred when amphiphilic molecules were incorporated in the outer monolayer of the erythrocyte membrane. Compounds penetrating to the inner monolayer of the membrane induced formation of stomatocytes. We can therefore assume that BCF and BCL extract components concentrated mainly in the outer monolayer of the erythrocyte membrane.

### 3.5. FTIR Investigation of Erythrocyte Membrane

**Figure 7:**
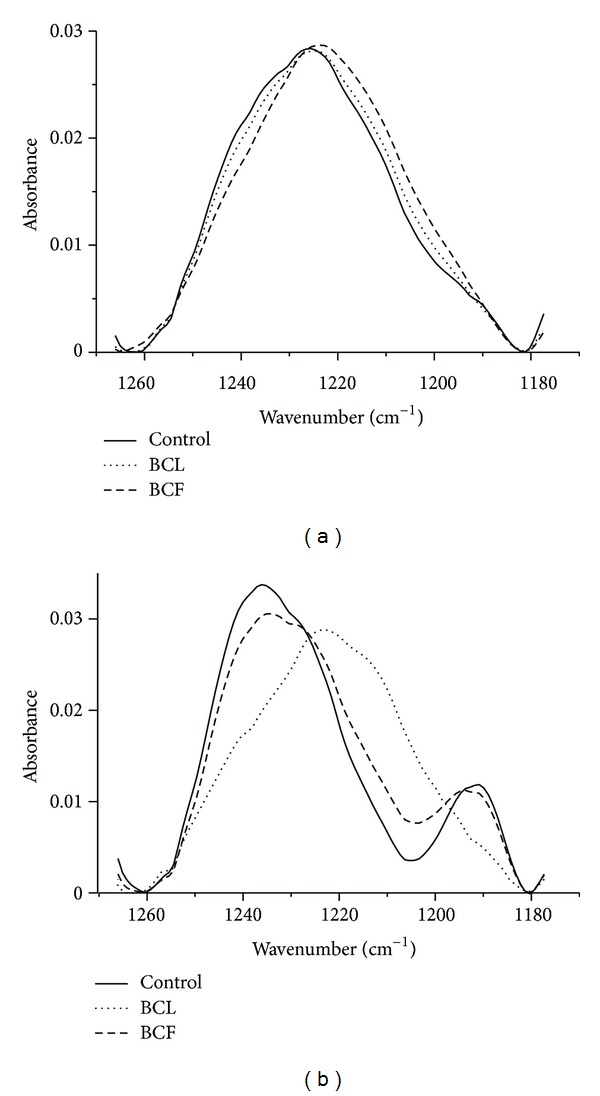
Phosphate band of erythrocyte membrane modified with BCF and BCL extract (a), and of erythrocyte membrane modified with BCF and BCL extract, irradiated with UVC (b). Solid line, control membrane; dashed line, membrane with fruit extract; dotted line, membrane with leaf extract.

Spectroscopic studies in the near infrared allowed to take absorption spectra for unmodified and BCL and BCF modified erythrocytes. Spectra of unmodified and extract modified membranes irradiated with UVC were also taken. The results obtained allowed us to observe changes induced in the erythrocyte membrane by UVC radiation and phenolic components of the extracts. In the spectra, the characteristic frequency bands of the protein and lipid components were analysed. We identified the characteristic for erythrocytes frequency bands for individual membrane components: methyl groups and methylene hydrocarbon chains (2980–2830 cm^−1^), carbonyl groups of lipids (1770–1710 cm^−1^), the amide band I (1690–1590 cm^−1^) and amide band II (1580–1500 cm^−1^), and phosphate band (1280–1200 cm^−1^) and choline band (1000–940 cm^−1^) [45–48]. Analysis of the spectra of membranes treated with aqueous extracts of blackcurrant leaves and fruit showed no significant changes in the amide I and amide II bands (vibration bands of groups C=O and N–H proteins). There were also no changes in the vibrational bands of methyl and methylene groups, alkyl chains of lipids, and C=O vibrations of carbonyl groups of lipids. This result suggests that the polyphenolic compounds contained in the extracts do not significantly affect the structure of the proteins contained in the membrane of red blood cells, and they do not modify the hydrocarbon chains of lipid molecules. UVC radiation exposure of control unmodified membranes and membranes modifies with the extracts does not cause visible changes in the above mentioned bands. The presence of the extracts in erythrocytes membranes causes a slight shift of the phosphate band in the direction of smaller wave numbers, which points to a slight increase in the degree of hydration of PO_2_ group. A slightly larger displacement of this band is caused by the extract from BCL. Exposure of the membranes to UVC radiation results in a significant shift of the phosphate band towards higher values of wave numbers, suggesting a clear reduction in the number of OH groups associated with lipid phosphate groups via hydrogen bonds. The presence of the extracts in a suspension reduced the transfer effect of phosphate bands distinctly. Phosphate bands of irradiated membranes in the presence of extracts are shifted toward lower wave numbers relative to the irradiated ghosts, which is particularly visible with BCL extract. So we can assume that the presence of extracts in membranes exposed to UVC protects them against changes induced by the radiation, causing the membrane properties becoming closer to those of membrane not exposed to radiation. A particularly high level of membrane protection was observed in the presence of blackcurrant leaf extract (Figure 7).

**Figure 8:**
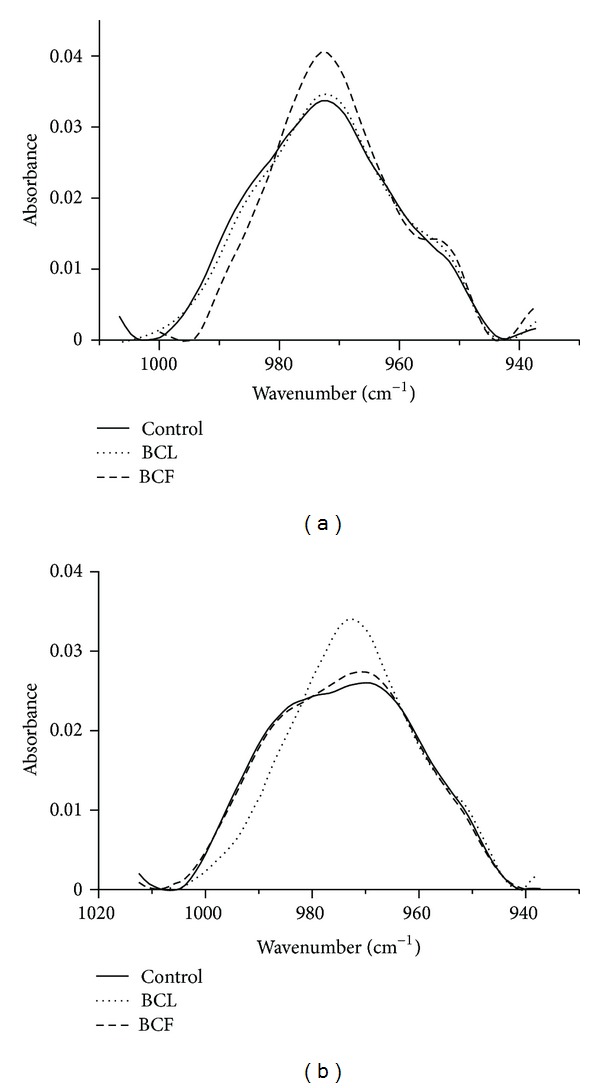
Choline band of erythrocyte membrane modified with BCF and BCL extract (a), and of erythrocyte membrane modified with BCF and BCL extract, irradiated with UVC (b). Solid line, control membrane; dashed line, membrane with fruit extract; dotted line, membrane with leaf extract.

The presence of extract and exposure to UVC light caused visible changes also in the choline N^+^ (CH_3_) band of lipids. In the case of ghosts not exposed to UVC the presence of extracts caused distinct decrease in the half-width of the choline band, which suggests a change in the packing order in that part of membrane and can be linked with presence of sugar groups in the extract components. UV radiation caused shift of the band towards lower wave numbers. For ghosts irradiated in the presence of fruit extract the effect of light was markedly smaller, while in the presence of leaf extract no changes were observed compared with nonirradiated ghosts (Figure 8).

### 3.6. Statistical Analysis

Statistical analysis was carried out using Statistica 9.0 (StatSoft Inc.). All the experiments were performed at least in triplicate unless otherwise specified. Analysis of variance was carried out and significance between means was determined using Dunnett's post hoc test. Results are presented as mean ± SD. Significant levels were defined at *P* < 0.05.

## 4. Discussion

The results of the presented research have shown that blackcurrant leaves (BCL) and blackcurrant fruits (BCF) extracts induce small changes in erythrocyte membranes. Their polyphenolic composition makes possible an interaction with the lipids of biological membranes. The main polyphenolic constituent of BCF extract are anthocyanins, which constitute over 98% of the total polyphenol content of the extract. It was shown that the beneficial properties of anthocyanins, cyanidins and their glycosides, are connected with their ability to scavenge free radicals, including the reactive forms of oxygen [49–51]. Cyanidins were found to bind with the biological membrane, protecting it against oxidation [21, 52–54]. It can thus be assumed that mainly anthocyanins are responsible for the protective action of blackcurrant fruit extracts with respect to the biological membrane.

BCL extract differs in polyphenolic composition from BCF extract, where quercetin derivatives dominate, which constitute ca. 80% of the total polyphenols content of the extract. An antioxidation activity of quercetin glycosides in relation to the erythrocyte membrane was shown in our previous studies [20]. However, it is not yet fully elucidated the molecular mechanism of the antioxidant action of these compounds in relation to the biological membrane.

The present study aimed to determine the antioxidative activity of blackcurrant extracts with respect to the membrane of red blood cells, treated as a model of the biological membrane. The impact of the extracts on erythrocyte membrane properties was also examined. Selected research methods helped to determine the location of the compounds contained in the extracts, based on erythrocyte shape changes and hemolytic and FTIR studies. The results of the investigations undertaken enabled us to determine the relationship between the antioxidative activity of the extracts and their affinity to the membrane.

Hemolytic tests showed that polyphenolic compounds contained in the extracts do not induce hemolysis, therefore do not cause lytic effect on red blood cells in the used range of concentrations. The lack of hemolysis and cytotoxicity for chosen flavonoids was also confirmed in the work [55]. One can therefore assume, on the basis of the studies [56–60], that the compounds do not embed deep into the hydrophobic membrane area. Hemolysis caused by various lytic compounds occurs when such substances penetrate deeply into the membrane, weakening the interaction between its components. Disturbed is the structure of the membrane and facilitated transport of water swells the cell, and the membrane is permanently damaged. Studies of osmotic resistance confirmed the hemolytic results, they showed that as a result of incorporation of the extract contained compounds the osmotic resistance does not change or is even greater in the case of BCL extract.

The extract from the fruit does not alter the osmotic resistance, that is, does not change the properties of the erythrocyte membrane, whereas the leaf extract causes an increase in the resistance, which means that substances present in the extract bind to the membrane and make it stronger, so that it becomes less sensitive to changes in osmotic pressure.

Both the extracts induced an alternation in the erythrocyte morphology, from normal discoid shape to an echinocytic form. BCF and BCL extracts are responsible for creation of varied forms of echinocytes, BCL mainly echinocytes and BCF echinocytes and spheroechinocytes. It can thus be assumed, according to the bilayer couple hypothesis [42], that the extracts concentrate mainly in the outer monolayer of the erythrocyte membrane when inducing various forms of echinocytes, and practically do not permeate into the inner monolayer of the membrane.

The changes observed in the IR spectrum suggest a superficial character of the interaction of BCF and BCL components with the hydrophilic part of the erythrocyte membrane. FTIR spectra for extract modified membranes showed no significant changes in the hydrophobic part of the hydrocarbon chains, but showed a change in the degree of hydration in the phosphate and choline band of membrane phospholipids. As the authors suggest [48, 61], as a result of the effect of polyphenols on lipid membrane, hydrogen bonds are formed between the hydroxyl groups of polyphenols and the polar groups of lipids. In addition, numerous studies showed that plant extracts, particularly those rich in polyphenol compounds with sugar residues, reduce the packing order of the hydrophilic area of membrane, causing increased hydration of this area.

As indicated by the results, after one hour of exposure to UVC radiation, changes occurred in the erythrocyte membrane, in particular in its hydrophilic region. This is shown by a clear shift of the phosphate band of polar heads of lipids, which may be related to oxidation of OH groups which are connected by hydrogen bonds with the phosphate groups or lipids. The presence of the extracts in ghosts suspension markedly reduced the effect of phosphate band transfer that was caused by exposure to UV radiation. In particular, such an effect was observed in the case of BCL extract, which caused a complete abolition of UVC induced changes in control ghosts, and even a visible widening of the band. This may suggest that during exposure to UVC radiation in the presence of the extracts (BCL and BCF) the membrane phosphate groups were not oxidized but had undergone hydration (BCL), which also indicates the presence of extract components in this part of the membrane. In addition, it is believed that the polyphenolic compounds, especially those occurring in the form of glycosides, cause hydration of the membrane, transporting numerous water molecules bound to them [31, 40, 48–51, 53, 54, 56–61]. Such protective action of the extracts can be attributed to their phenolic components that bind to the membrane surface, and containing numerous OH groups in their structure they, possibly, as the first undergo oxidation.

The location of the compounds in the hydrophilic part of membrane seems to constitute a protective shield of the cell against other substances, the reactive forms of oxygen in particular, which finds its reflection in their antioxidant properties. BCL and BCF extracts exhibited a high antioxidant activity towards reactive forms of oxygen, which developed as a result of membrane photo-oxidation induced by UVC radiation and by the AAPH compound.

## 5. Conclusion

The results obtained indicate that BCL and BCF extracts exert beneficial effect on the organism protecting the cell membrane against oxidation. The polyphenolic compounds contained in the extracts do not disturb the structure of the biological membrane, to which they bind only superficially, penetrating its hydrophilic region, and do not penetrate the membrane hydrophobic region, as is evident from the hemolytic, microscopic, and FTIR investigations. It can thus be expected that they do not disturb the function of the biological membrane.

In this context, a protection of the organisms against the harmful effects of free radicals and reactive oxygen species is made possible by providing the body with appropriate quantities of plant polyphenolic substances. Thus, the results of this research encourage the consumption of fruits and extracts from blackcurrant leaves to fight free radicals and thus increase our resistance to many diseases.

## Figures and Tables

**Table 1:** The percent content and characterization of phenolic compounds of the extract of blackcurrant fruits (BCF) and leaves (BCL) using their spectral characteristic in HPLC-DAD (retention time, *λ*_max_) and positive and negative ions in UPLC-ESI-MS [M–H]^−^.

Compounds	BCF	BCL*	BCF/BCL	BCF/BCL	BCF/BCL
	Content (%)		*R*_*t*_ (min)	*λ*_ max_ (nm)	(MS) (m^−^)
Chlorogenic acid	0.12	1.15	10.71	320	353
p-Coumaroylquinic acid	0.11	0	7.18	311	341
Neochlorogenic acid	0	0.1	7.18	320	353
Cryptochlorogenic acid	0	0.11	10.87	320	353
Delphinidin-3-O-glucoside	4.58	0	15.29	526	465
Delphinidin-3-O-rutinoside	22.49	0	16.21	525	611
Cyanidin-3-O-glucoside	6.00	0	17.03	516	449
Cyanidin-3-O-rutinoside	21.18	0	18.03	516	595
Petunidin-3-O-rutinoside	0.24	0	19.17	527	625
Quercetin-3-O-rutinoside	0.11	0.33	23.17/19.04	355	611/609
Quercetin-3-O-galactoside	0.17	2.52	23.77/20.71	355	465/463
Quercetin-3-O-glucoside	0.15	0	24.46	355	465
Quercetin-3-(6′′-malonyl)-glucoside	0	1.91	21.29	355	549
Quercetin-3-O-glucosyl-6′′-acetate	0	5.53	23.17	354	505
Myricetin-3-O-rutinoside	0.04	0	19.04	355	627
Myricetin-3-O-galactoside	0.88	0	20.71	358	481
Myricetin-3-O-glucoside	0.08	0	21.29	356	481
Kaempferol-3-O-rutinoside	0	0.23	23.77	342	593
Kaempferol-3-O-galactoside	0	0.41	24.46	342	447
Kaempferol-3-O-glucosyl-6′′-acetate		0.96	26.96	346	489

Total	56.14	13.23			

*Published previously in Oszmiański et al., 2011 [34].

**Table 2:** Values of IC_50_ for BCL and BCF extracts, and Trolox that inhibit erythrocyte membrane lipids oxidation by 50%, determined with the fluorimetric method. The oxidation was induced with UVC radiation and the AAPH compound.

Extract/inducer	Concentration IC_50_ (*µ*g/mL) ± SD	
	UVC	AAPH
Blackcurrant leaves (BCL)	24.5 ± 1.7	7.2 ± 0.05
Blackcurrant fruits (BCF)	25.6 ± 1.5	4.8 ± 0.16
Trolox	14.6 ± 1.3	3.9 ± 0.30

**Table 3:** Percent content of hemolyzed erythrocytes in the presence of BCL and BCF extracts of specific concentrations.

Extracts	Percentage of hemolysis ± SD	
	Blackcurrant	
Concentration (mg/mL)	BCF	BCL
Control	5.10 ± 0.56	5.10 ± 0.56
0.01	0.97 ± 0.11	0.98 ± 0.11
0.02	2.18 ± 0.26	1.60 ± 0.19
0.03	2.82 ± 0.34	2.01 ± 0.24
0.04	4.39 ± 0.53	2.50 ± 0.30
0.05	5.22 ± 0.63	3.38 ± 0.40
0.06	5.23 ± 0.63	4.22 ± 0.51
0.07	6.18 ± 0.74	5.14 ± 0.62
0.08	6.89 ± 0.83	5.95 ± 0.71
0.09	7.41 ± 0.89	7.69 ± 0.92
0.10	8.70 ± 1.05	9.41 ± 1.13
